# The Role of Gasdermin-D-Mediated Pryoptosis in Organ Injury and Its Therapeutic Implications

**DOI:** 10.1080/15476278.2023.2177484

**Published:** 2023-02-22

**Authors:** Joud Mulla, Rohan Katti, Melanie J. Scott

**Affiliations:** Department of Surgery, University of Pittsburgh, Pittsburgh, Pennsylvania, USA

**Keywords:** Gasdermin-D, GSDMD, organ injury, pyroptosis, inflammsome

## Abstract

Gasdermin-D (GSDMD) belongs to the Gasdermin family (GSDM), which are pore-forming effector proteins that facilitate inflammatory cell death, also known as pyroptosis. This type of programmed cell death is dependent on inflammatory caspase activation, which cleaves gasdermin-D (GSDMD) to form membrane pores and initiates the release of pro-inflammatory cytokines. Pyroptosis plays an important role in achieving immune regulation and homeostasis within various organ systems. The role of GSDMD in pyroptosis has been extensively studied in recent years. In this review, we summarize the role of GSDMD in cellular and organ injury mediated by pyroptosis. We will also provide an outlook on GSDMD therapeutic targets in various organ systems.

## Overview of pyroptosis and gasdermin-D

Pyroptosis is an inflammatory form of programmed cell death mediated by inflammatory caspases upon exposure to a pathogenic infection, pathogen-associated molecular patterns (PAMPs), or host-derived damage-associated molecular patterns (DAMPs) through canonical and noncanonical pathways.^1–5^ The canonical pathway depends on the inflammasomes, such as NLRP1b, NLRP3, NLRC4, AIM2, or Pyrin, to form a complex and induce inflammation and systemic immune response by activating caspase-1. The noncanonical pathway is inflammasome-independent and occurs when gram-negative bacterial lipopolysaccharide (LPS) can recognize caspase-11 in mice and caspase-4/5 in humans. Gasdermin-D is a substrate of both caspase-1 and caspase-11, where they both cleave the N-terminal of Gasdermin-D (GSDMD-NT) to release pro-inflammatory cytokines interleukin-1β (IL1β) and interleukin-18 (IL18) which induce pyroptosis.^1–5^

Gasdermin (GSDM) is a family of proteins comprised six members: GSDMA, GSDMB, GSDMC, GSDMD, GSDME/DNFA5, and PVJK/GSDMF.^6^ They are mostly composed of C-terminal, N-terminal and linker domains.^7^ The first GSDM protein was discovered in 1998 as a mutation in age-related hearing loss as DNFA5.^6, 7^ In 2000, Saeki et al.^8^ identified GSDM gene expressed in GI tract and skin on mouse chromosome 11.^7–9^ GSDMD was determined to be required for pyroptosis in 2015.^10–12^ Shi et al.^10^ showed that cleavage of GSDMD by caspase-1 and other inflammatory caspases can drive pyroptosis.

However, Kayagaki et al.^11^ refuted Shi et al.^10^ hypothesis and demonstrated that caspase-11 requires GSDMD to promote pyroptosis, caspase-1 activation and non-canonical inflammasome signaling due to LPS.^10^ Upon proteolytic cleavage of GSDMD, the N-terminal fragment interacts with the membrane lipids to form GSDMD pores and promotes the secretion of IL-1βin pyroptosis, thus GSDMD is best known as a pyroptosis executioner.^12^

### The role of gasdermin-D in liver injury

#### Gasdermin-D-mediated pyroptosis is hepatoprotective in various NAFLD stages

Liver injury is the primary cause of abdominal trauma involving cell death and inflammation.^13,14^ GSDMD plays a major role in hepatocyte pyroptosis, with extensive studies done on its role in alcoholic fatty liver disease and various forms of liver injuries.^14–19^ Xu et. al, demonstrated that cleaved GSDMD N-terminal fragments are increased in both nonalcoholic fatty liver disease (NAFLD) and nonalcoholic steatohepatitis patients (NASH), resulting in lobular inflammation, which is one of the key features of NASH.^15, 16, 18^ As a result, several studies show that inhibiting hepatocyte GSDMD-mediated pryoptosis can alleviate different severities on liver injuries.^17–21^ Yin et al.^17^ demonstrate that Jiangzhi Ligan Decoction (JZLGD), a Chinese herbal formula, decreased hepatic steatosis and liver inflammation by inhibiting GSDMD levels and its N-terminal fragments, by preventing the expression of ASC, NLRP3, Caspase-1 and Caspase-11 located upstream of GSDMD in both canonical and non-canonical pathways. JZLGD regulates the activation of GSDMD, pore formation, and the release of proinflammatory cytokines IL1β and IL-18.^17^

GSDMD has also been shown to target different levels of NAFLD such as fibrosis by activating stellate cells.^19^ This indicates that regulation of hepatocyte pryoptosis can lead to hepatoprotective effects in different NAFLD stages. Other studies investigated the effect of inhibiting hepatocyte pyroptosis in severe forms of liver injury. Wang et al.^20^ show that phenethyl isothiocyanate (PEITC), a natural product found in cruciferous vegetables, can significantly reduce both chemical and inflammatory liver injury. Similar to JZLGD, PEITC targets NLRP3 and Caspase-1. Additionally, PEITC directly inhibits GSDMD cleavage and membrane pore formation by binding to cysteine 191 on GSDMD.^20^

#### Gasdermin-D-mediated pyroptosis plays a role in hepatoxicity and liver failure

As acute liver injury (ALI) may progress to acute liver failure (ALF), a recent study demonstrated Necrosulfonamide, a GSDMD inhibitor, to alleviate ALF induced by Lipopolysaccharide/D-galactosamine Table 1.^21, 33^

**Table 1.** Summary of therapeutic compounds in organ injury targeting pathways leading to GSDMD-mediated pyroptosis.

Organ injury	Disease	Therapies	Target	Reference
Liver	NAFLD/NASH	Jiangzhi Ligan Decoction (JZLGD)	ASC, NLRP3, Caspase-1 and Caspase-11 located upstream of GSDMD	K. Yin et al.^17^
Central nervous system	Post-ischemic stroke	VX765	Caspase-1	J Li et al. ^22^
	Spinal cord injury	Carbon monoxide release molecule-3 (CORM3)	Inflammasome	J. P. de Rivero Vaccari et al.^34^
Cardiovascular	myocardial ischemia/reperfusion injury	Emodin	TLR4/MyD88/NkFB/NLRP3/GSDMDM pathway	B. Ye et al ^23^
	Abdominal aortic aneurysm	Difluoromethylornithine (DMFO)	Putrescine synthesis	J. Goa et al. ^24^
Lung	Sepsis-induced acute lung injury	Hemin	NLRP3/ASC/Caspase-1	Y. P. Luo et al. ^25^
	Sepsis-induced acute lung injury	Corticosteroids	1) NFkB signaling pathway 2) mitochondria ROS dependent NLRP3 activation	J. Yang et al. ^26^
	Sepsis-induced acute lung injury	Cyclic helix B peptide	NLRP3, IL1B secretion	P. X. Zhang et al. ^27^
	brain injury induced by acute lung injury	Ghrelin	TLR4-NFκB pathway	F X Shao et al. ^28^
	Lung vaso-occlusion in sickle cell disease	Z-VAD-FMK	Caspase inhibitor	R. Vats et al. ^29^
Kidney	Renal Ischemia-Reperfusion Injury	Parthenolide	NFκB and Tisp40	C. Xiao et al. ^30^
Gastrointesinal tract	DSS-induced colitis	cGAS inhibitor RU.521	cGAS signaling pathway	C. Ma et al. ^31^
	Ulcerative colitis	Honokiol	TLR4/NFκB	N. Wang et al. ^32^

GSDMD inhibition has been shown to be hepatoprotective in NAFLD, ALI and ALF. However, additional studies show GSDMD deficiency can be hepatotoxic in noninfectious liver injury such as Acetaminophen (APAP)-induced liver failure and hypoxic liver induced by hemorrhagic shock.^35^ Yang et al.^35^ demonstrated that inhibiting GSDMD in mice increases liver damage after APAP overdose and hemorrhagic shock due to increase in hepatocyte death. When GSDMD is inhibited, caspase-8 cleavage is increased, resulting in increased apoptosis and necroptosis and ultimately hepatocyte death.^35^ Activation of GSDMD is hepatoprotective in noninfectious liver injury by downregulating caspase-8 cleavage, resulting in reduction of apoptosis and necroptosis signaling pathways.^35^ They further showed that inhibition of GSDMD leads to absence of pyroptosis resulting in the activation of other cell death signaling pathways such as apoptosis and necroptosis.^35^ In line with their study, Yang et al.^35,^ also shed light on hepatocyte morphology when undergoing GSDMD-mediated pyroptosis. Pyroptosis in hepatocytes exhibit low levels of GSDMD cleavage compared to macrophages. Hepatocytes are also shown to be resistant to pyroptosis by experiencing slight cell shrinkage and no cell rupture.^36^

GSDMD-mediated pyroptosis plays a role in 70% partial hepatectomy (PH), where its inhibition alleviates liver injury and accelerates liver regeneration through upregulation of mitogen signaling pathways, including epidermal growth factor receptor (EGFR) and hepatocyte growth factor receptor (HGFR) pathways.^37^

#### Gasdermin-D is essential in Kupffer cells and liver sinusoidal endothelial cells

There are few studies investigating the role of GSDMD in hepatic stellate cells, liver sinusoidal endothelial cells and Kupffer cells. Yamagishi et al.^38^ demonstrated that GSDMD-NT cell membrane pore formation mediates the release of IL-33 from senescent hepatic stellate cells, which promotes obesity-associated hepatocellular carcinoma. Interestingly, one study showed that GSDMD specific deficiency in myeloid cells is protective hepatic-ischemia reperfusion injury (IRI), with no protective effects in GSDMD specific deficiency in hepatocytes cells.^39^ Lastly, GSDMD plays a crucial role in disseminated intravascular coagulation (DIC) in sepsis; GSDMD deficiency ameliorates thrombin generation, fibrin deposition, and platelet aggregation in the liver microvasculature.^40^

GSDMD is a double-edged sword, since it is involved in different cell death signaling pathways, its effect on liver injury depends on the severity of the damage.^36^ We can conclude that GSDMD inhibition is hepatoprotective due to the rigorous research on severe liver injury and various liver cells. GSDMD deficiency in hepatoprotective in various liver injuries includes, but is not limited to, NAFLD, ALI, ALF, and 70% PH. In addition, GSDMD deficiency further inhibits DIC in sepsis in the liver microvasculature and protects against obesity-induced HCC^38,40^. However, further studies are needed to determine how GSDMD-mediated pyroptosis leads to liver injury and disease and investigate its effect in different liver cell types, including stellate and parenchymal cells.

### The role of gasdermin-D in brain injury

#### Gasdermin-D plays a role in Ischemia/reperfusion brain injury

Central nervous system injuries are common causes of morbidity and mortality at different ages, lacking sufficient treatments.^41^ GSDMD-mediated pyroptosis is associated with traumatic brain and spinal cord injuries, where recent studies and reviews highlight the role of NLRP3 in the central nervous system.^42–45^ However, in the past five years, GSDMD has been extensively studied in ischemic stroke and cerebral ischemia, making it a potential therapeutic target in ischemia and reperfusion brain injury.^22, 41, 46^ Zhang et al.^46^ showed that middle cerebral artery occlusion/reperfusion has elevated levels of GSDMD and N-terminal fragments (GSDMD-NT) that induce pyroptosis. In addition, another group demonstrated that pyroptosis occurs in neurons.^22^ They showed significantly high GSDMD expression on day one post-acute stroke.^22^ They further assessed the neuronal effects of VX765 treatment, a caspase-1 inhibitor, post-ischemia, which showed a significant reduction of GSDMD on day one and suppression of the canonical inflammasome pathway leading to neuroprotection following ischemic stroke.^22^ GSDMD-mediated pyroptosis was also shown in microglia after ischemia/reperfusion brain injury. Therefore, knocking down GSDMD improves recovery after ischemic brain injury by preventing pro-inflammatory cytokine release of IL-1β and IL18.^41^

#### Inflammasome inhibition regulates GSDMD-mediated pyroptosis in brain injury

Interestingly, growing evidence documents the effect of AIM2 inflammasome on GSDMD-mediated pyroptosis in early brain injury (EBI) after aneurysmal subarachnoid hemorrhage (SAH).^47^ EBI after SAH involve the secretion of pro-inflammatory cytokines IL-1β and IL-18. Patients with SAH exhibit high levels of AIM2 inflammasome in their cerebrospinal fluid (CSF).^47^ Since AIM2 is upstream to GSDMD in the canonical pathway, they revealed that GSDMD-mediated pyroptosis by AIM2 inflammasome is essential in EBI after SAH.^47^ These findings suggest potential therapeutic targets for different brain injuries by regulating GSDMD-mediated pyroptosis.

#### Inflammasome inhibition regulates GSDMD-mediated pyroptosis in spinal cord injury

GSDMD-mediated pyroptosis can further regulate spinal cord injuries (SCI). Inflammasomes play a critical role in SCI, resulting in inflammatory cell death.^48, 49^ Recent evidence demonstrated the therapeutic effects of inhibiting GSDMD and suppressing pyroptosis in SCI to reduce the inflammatory response.^47, 34,50–52^ Dai et al.^50^ demonstrated that ASC, caspase-1, and GSDMD in the canonical inflammasome pathway associated with pyroptosis, were significantly elevated in spinal cord injury rat models compared to the sham groups. They inhibited SCI pyroptosis and neuroinflammatory response by using Celastrol, a therapeutic agent for cerebral ischemia, significant neuroinflammation reduction seven days after spinal cord injury in rat models and recovery of motor dysfunction.^50^

Another group further investigated the effect of CD73, an immune homeostasis regulator, in GSDMD-mediated pyroptosis.^44, 51^ S. Xu and colleagues^51^ first showed that CD73 and GSDMD levels are positively correlated with the severity of SCI patients.They further elucidated the CD73 mechanism in microglia pyroptosis, where CD73 overexpression increased Foxo1 activation in PI3K/AKT/Foxo1 in BV2 microglial cells.^51, 52^ Interestingly, they revealed that Foxo1 is a transcriptional activator in the promoter region of the GSDMD gene, indicating that CD73 regulates the expression of GSDMD through Foxo1.^51, 52^ CD73 targeting GSDMD through PI3K/AKT/Foxo1 mechanism is a promising approach for identifying an effective therapeutic target for SCI. In addition, AIM2 inflammasome has also been shown to play a role in SCI.^53^ Using Carbon monoxide release molecule-3 (CORM3) has been shown to inhibit inflammasome activation, which is a potential therapeutic target for SCI.^53^ Nevertheless, further studies need to investigate the potential role of GSDMD-mediated pyroptosis by AIM2 inflammasome in SCI (Table 2).

**Table 2.** Summary of therapeutic compounds in organ injury targeting GSDMD cleavage, IL1β and IL18 release.

Organ injury	Disease	Therapies	Target	Reference
Liver	Severe liver injury	isothiocyanate (PEITC)	NLRP3, Caspase-1, Cystiene 191 of GSDMD	J. Wang et al. ^20^
	Acute liver failure induced by Lipopolysaccharide/D-galactosamine	Necrosulfonamide	GSDMD	Wu et al. ^21^
Central nervous system	Acute spinal cord injury	Celastrol	NLRP3, ASC, Caspase-1, GSDMD	W. Dai et al. ^50^ Xu et al. ^51^
Cardiovascular	Acute Myocardial injury	Necrosulfonamide	GSDMD	Wu et al. ^21^ K.Jiang et. al ^54^
	Ischemic stroke	DL-3-n-butylphthalide (NBP)	GSDMD-NT	B. Han et al. ^55^
Lung	Sepsis-induced acute lung injury	Dihydromyricetin (DHM)	NLRP3/caspase-1/IL1β/IL18/GSDMD-NT	Yc. Wang et al. ^56^
	Severe acute pancreatitis (SAP)-associated lung injury	Disulfiram	GSDMD, IL1β and IL18 release	J. Wu et al. ^57^
	SAP in intestinal injuries	siRNA-GSDMD	GSDMD, IL1β and IL18 release	T. Lin et al. ^58^
	Multiple organ dysfunction syndromes	Disulfiram	GSDMD, IL1β and IL18 release	C. Silva et al. ^59^
	Lung vaso-occlusion in sickle cell disease	Necrosulfonamide, Disulfiram, LDC7559	GSDMD-NT	R. Vats et al. ^29^
Kidney	Cisplatin-induced acute kidney injury	Paricalcitol, a vitamin D receptor agonist	NFκB/NLRP3/cleaved caspase-1/GSDMD	S. Jiang et al. ^60^
	Iopromide-induced AKI	Acetylbritannilactone	NLRP3/ASC/GSDMD and release of IL1β and IL18	F. Chen at al. ^61^

### The role of gasdermin-D in cardiovascular Injury

Emerging evidence suggests the role of GSDMD in cardiomyocytes pyroptosis. Lei and colleagues revealed the role of pyroptosis and oxidative stress in myocardial infarction (MI), where they examined GSDMD and its transcription factor NFκB.^62^ Using the myocardial infarction rat model, they revealed that GSDMD-mediated pyroptosis by NLRP3 inflammasome results in cardiomyocyte loss following MI. They further showed that inhibiting oxidative stress can reduce NLRP3-mediated pyroptosis and therefore regulate the activity of the NFκB-GSDMD signaling axis, making it a promising approach for targeted MI therapies.^62^ GSDMD plays a role in different stages of MI. MI can result in ischemic myocardial tissue, and myocardial ischemia/reperfusion (I/R) injury upon reperfusion. Shi and colleagues^63^ investigated the role of GSDMD-mediated cardiomyocyte pyroptosis in MI I/R injury, where they proved that GSDMD deficiency reduced myocardial I/R in mice. In addition, they demonstrated that GSDMD was cleaved in cardiomyocytes when stimulated with H_2_O_2_ to induce oxidative stress.^63^ GSDMD deficiency has also been shown to attenuate LPS-induced septic myocardial dysfunction by reducing cardiac inflammation, NFκB signaling pathways, ROS production and NLRP3 activation.^64^

#### NFkB/NLRP3/GSDMD pathway is a therapeutic target in myocardial infarction (MI)

In line with the previous study, several studies revealed GSDMD to be a potential therapeutic target in MI and myocardial I/R injury through NFκB/NLRP3/GSDMD pathway. Ye and colleagues^23^ showed that mRNA levels of GSDMD and its N-terminus (GSDMD-NT) were upregulated in cardiomyocytes after hypoxia/reoxia (H/R). They investigated the effects of an anti-inflammatory compound, Emodin, which protected cardiomyocytes from GSDMD-mediated pyroptosis when exposed to H/R.^23^ Emodin showed a protective mechanism by reducing infarct size after myocardial I/R injury and pyroptosis in vivo. They further elucidated the Emodin mechanism, which targets the TLR4/MyD88/NFκB/NLRP3 pathway, all leading to GSDMD cleavage and pyroptosis.^23^ TLR4 recruits MyD88, which triggers NFκB activation, a GSDMD transcription factor.^23, 63, 64^ NF κ B activates NLRP3, resulting in the cleavage of caspase-1 and ultimately GSDMD cleavage.^23, 65^ Conversely, Emodin blocks the TLR4 pathway, inhibiting NFκB and NLRP3 activation, caspase-1 and GSDMD cleavage, and IL1βrelease.

#### Gasdermin-D-mediated pyroptosis is activated in acute myocardial infarction (AMI)

In more recent studies, GSDMD is also shown to be activated in acute myocardial infarction (AMI), indicating that pyroptosis plays an essential role in the progression of AMI.^54, 66^ Zhang et al.^66^ revealed that pro-inflammatory adipokine, retinol-binding protein 4 (RBP4), contributes to AMI by activating the canonical inflammasome NLRP3/Caspase-1/GSDMD-NT resulting in pyroptosis. Furthermore, they showed that knockdown of RBP4 can attenuate NLRP3-mediated pyroptosis and protect against cardiac dysfunction after AMI injury. Thus, targeting RBP4 can be beneficial for AMI treatment.^66^ These studies highlighted the importance of NFκB/NLRP3/GSDMD pathway in regulating cardiac dysfunction and myocardial injury. K. Jiang and colleagues^54^ further demonstrated that GSDMD activation in AMI injury results in infiltration of neutrophils/monocyte and cardiac inflammation. In groundbreaking results, Jiang et al.^54^ revealed that GSDMD pharmacological inhibition using Necrosulfonamide can reduce infarct size after AMI injury (Table 1).

#### Gasdermin-D-mediated pyroptosis plays a major role in endothelial dysfunction

Pulmonary Arterial Hypertension (PAH) is caused due to endothelial dysfunction, which enhances vascular inflammation due to activation of caspase-1 and caspase-11 in pyroptosis.^67^ Caspase-11 in mice, homologous to caspase-4 in humans, was activated in PAH rat models and injury simulated human pulmonary artery endothelial cells (HPAECs) by TNF α.^67^ They inhibited caspase-11/4 activity using wedelolactone, which hindered the progression of PAH rat models.^67^ Wu and colleagues^67^ further examined downstream effects of caspase-11 in TNF α-induced HPAECs, which activated GSDMD to induce endothelial cell pyroptosis. GSDMD is activated through different inflammasome pathways; therefore, GSDMD inhibition in PAH needs to be further studied and validated.

NLRP3/GSDMD has been further studied in coronary endothelial cell dysfunction in Kawasaki disease (KD).^68^ KD is the most common cause of pediatric cardiac disease in developed countries.^68^ In addition to coronary endothelial cell dysfunction, KD is also caused by the production of pro-inflammatory cytokine IL1β; thus, the role of pyroptosis was investigated in endothelial cells in KD.^68^ Jia and colleagues^68^ revealed that proteins in the canonical inflammasome pathway associated with pyroptosis (ASC, cleaved caspase-1, GSDMD, matureIL1β, and IL18) are significantly elevated in KD patients compared to healthy personnel. Moreover, NLRP3 inflammasome was expressed in KD-treated endothelial cells, resulting in activation of downstream NLRP3-mediated pyroptosis, including cleaved caspase-1 GSDMD-NT, mature IL1β, and IL8.^68^ These results suggest the role of endothelial cell pyroptosis in KD and how targeting NLRP3-mediated pyroptosis, including GSDMD-NT, could be a potential targeted therapy for KD and other related conditions with systemic inflammation.

#### GSDMD-mediated pyroptosis effects vascular inflammation

As myocardial hypertrophy is associated with chronic inflammation and increase in inflammatory cytokines, GSDMD-mediated inflammation also plays a role in myocardial hypertrophy and cardiac dysfunction.^55, 69, 70^ Han and colleagues^55^ investigated the effects of using DL-3-n-butylphthalide (NBP), a neuroprotective agent widely used in various Asian countries to treat ischemic stroke, in GSDMD-mediated inflammation. They used a transverse aortic constriction (TAC) mouse model to induce cardiac injury, which showed that NBP administration prevents myocardial hypertrophy and cardiac dysfunction by targeting GSDMD-NT and reducing inflammation.^55^ GSDMD has been studied in various cardiac dysfunction and vascular inflammation severities. Cardiovascular endothelial cell injury induced by Decabromodiphenyl ethane (DBDPE) enhances NLRP3 and caspase-1 activity, yet no studies have covered the therapeutic implications of GSDMD in cardiovascular injury induced by air pollutants.^71^ Lastly, GSDMD plays a role in vascular smooth muscle cells and cardiovascular injury. GSDMD specific deficiency in vascular smooth muscle cells alleviates abdominal aortic aneurysm (AAA) by reducing putrescine compound levels in the aorta.^24^ Inhibiting the synthesis of putrescine with difluoromethylornithine (DMFO), a compound in a clinical trial for Neuroblastoma, results in the prevention of AAA development. Thus, DMFO could be a potential drug for AAA treatment with few side effects (Table 1).^24^ GSDMD is activated in macrophages and vascular smooth muscle cells in human plaques, which exacerbates atherogenesis; thus, inhibition of GSDMD and pyroptosis in atherosclerosis can be a potential therapeutic target.^72^

### The role of gasdermin-D in lung injury

GSDMD plays a pivotal role in circulating vesicles in endothelial cell lung injury including sepsis-mediated pulmonary vascular endothelial cell injury and ventilation-induced lung injury.^73, 74^ During a pathological condition of the vascular endothelium, microparticles encapsulating caspase-1 are being released, where they circulate and accumulate in areas of disordered blood flow.^73, 75, 76^ Mitra et al.^73^ revealed that GSDMD induced by LPS simulation is encapsulated with the active caspase-1 microparticles to induce endothelial cell death. The group further showed that GSDMD knockout cells exhibit no circulation of active caspase-1 microparticles.^73^ Interestingly, a more recent group examined the effect of pyroptosis on circulating extracellular vesicles (EVs) in ventilation-induced lung injury (VILI).^76^ VILI has been shown to activate caspase-1/GSDMD in the lung, where only caspase-1 is transported to the brain in circulating EVs, leading to neuroinflammation and cell death by activating GSDMD and more caspase-1 in the brain.^74^ These studies highlight the importance of understanding circulating vesicles regulation in endothelial lung injuries inducing inflammation through GSDMD activation.

#### Gasdermin-D inhibition is a therapeutic target in lung inflammation

Many studies in the past few years investigated the role of LPS-induced lung vascular endothelial pyroptosis leading to acute lung injury and ultimately traumatic brain injury and multiorgan failure dysfunction.^77–80^ These studies primed the way to explore different therapeutic targets of acute lung injury and lung inflammation (Table 1 & Table 2). Wang and colleagues^56^ examined the effect of Dihydromyricetin (DHM), an anti-inflammatory flavonoid, in the cecal-ligation puncture model of sepsis to induce acute lung injury, which showed significant downregulation of lung inflammation by targeting the NLRP3/caspase-1/IL1β/IL18/GSDMD-NT pyroptosis pathway. DHM has been also previously shown to inhibit vascular endothelial cell pyroptosis *in vitro* through the Nfr2 signaling pathway.^81^ Downregulation NLRP3 inflammasome-mediated pyroptosis revealed many therapeutics approaches in the treatment of sepsis-induced acute lung injury using hemin,^25^ corticosteroids,^26^ and cyclic helix B peptide.^27^ In addition, Geranylgeranyl pyrophosphate synthase large subunit (GGPPS1) has also been shown to be effective against sepsis-induced acute lung injury by suppressing NLRP3 inflammasome activity in the TLR4-NFκB pathway.^82, 83^ Consistent with the previous finding, Ghrelin, a hormone protective against neuronal injury and stroke, has been shown to alleviate brain injury induced by acute lung injury by blocking the TLR4-NFκB signaling pathway.^28^ Therapeutic targets in LPS-induced acute lung injury through pyroptosis have been heavily studied, showing promising results in inhibiting lung injury and pulmonary inflammation (Table 1).

#### Gasdermin-D inhibition impedes multiorgan failure development in sepsis

Furthermore, pancreatitis can induce lung injury, known as severe acute pancreatitis (SAP)-associated lung injury.^57^ Wu and colleagues^57^ revealed activation of GSDMD and release of IL1β and IL18 in SAP mice models. They further showed that Disulfiram, an approved drug for alcohol use disorder, prevented GSDMD activation and IL1β and IL18 release, ameliorating SAP-induced lung injury.^57^ GSDMD downregulation is also effective against SAP in intestinal injuries by reducing systemic inflammatory response.^58^ Intravenous injection of siRNA to deplete GSDMD in SAP mice models, reduced IL1β and IL18 levels and improved intestinal musical changes and intestinal villus breakage.^58^ These studies suggest that GSDMD inhibition can reduce systemic inflammation in multiple organ dysfunction syndromes studied by C. Silva and colleagues.^59^ Similar to Wu et al.'s findings,^57^ they showed effective treatment with Disfulram, which inhibited sepsis development to multiple organ dysfunction syndromes (Table 1).^59^

#### Gasdermin-D inhibition alleviates vaso-occlusion in sickle cell disease

Lastly, GSDMD plays a vital role in inflammatory lung injury in sickle-cell disease (SCD), which promotes caspase-4/11 dependent activation of neutrophil-GSDMD and shedding of neutrophil extracellular traps (NETs) in the liver in P-selectin dependent manner.^29^ The NETs translocate to the lung and lead to neutrophil-platelet aggregation.^29^ Vats et al.^29^ further showed that inhibition of GSDMD using GSDMD-NT inhibitors Necrosulfamide and LDC7559, pan-caspase inhibitor Z-VAD-FMK^38^ alleviated lung vaso-occlusion in SCD.

### The role of gasdermin-D in gastrointestinal tract injury

#### Gasdermin-D is expressed in intestinal epithelial cells

GSDMD-associated pyroptosis contributes to inflammatory bowel disorders such as ulcerative colitis (UC) and Crohn’s disease and is increased in intestinal epithelial cells during colitis and inflammatory bowel disease (IBD).^84, 85^ GSDMD can be derived from dysregulated gut microbiota, specifically E.coli, increasing IL18 release, while mediating the release of nonlytic IL1β-containing small extracellular vesicles from the intestinal epithelial cells to promote DSS-induced colitis.^84, 85^ GSDMD also plays a role in metabolic diseases, systemic endotoxemia, and gut dysbiosis.^86^ Mice fed with high fat diet (HFD) have activated GSDMD-NT in mouse liver, kidney, and adipose tissue, and prevents systemic endotoxemia by killing endogenous bacteria (Proteobacteria) produced from LPS in HFD-mice.^86^ Interestingly, GSDMD is the only GSDM protein that can protect against *Salmonella typhimurium* gut infection.^86^

In addition to its activation in intestinal epithelial cells, GSDMD is also activated in colonic macrophages independent of the gut microbiota, making it protective in DSS-induced colitis.^31^ GSDMD^−/−^ mice treated with DSS exhibit more colitis phenotype, including shorter colon length and more body weight loss on day 9 compared to WT mice by exacerbating cGAS inflammation.^31^ The use of cGAS inhibitor RU.521 in the DSS-treated mice attenuates colitis phenotypes in WT mice but completely abolishes them in GSDMD^−/−^ mice, making it a potential target for protection from IBD.^31^

#### Gasdermin-D mediated macrophage pyroptosis plays a role in inflammatory bowel disorders

Another study targeted GSDMD-mediated pyroptosis in macrophages through TLR4/NFκB signaling pathway in ulcerative colitis using Honokiol, a compound isolated from genus *Magnolia*.^32, 87^ Wang et al.^32^ demonstrated that Honokiol targets the TLR4/NFκB suppressing gasdermin-D mediated pyroptosis through anti-inflammatory effects on DSS-induced colitis mice and LPS-induced RAW264.7 macrophages, making Honokiol a promising drug for UC.^32^ Lastly, GSDMD deficiency plays a role in gastric cancer (GC), where Wang et al.^88^ showed GSDMD downregulation in mouse GC tissue and human cell lines. GSDMD protects against gastric cancer development by inhibiting the S/G2 cell cycle and abnormal activation of the oncogenic signaling pathway.^88^

#### Gasdermin-B regulates inflammatory bowel disorders

Both Gasdermin-B (GSDMB) and Gasdermin-C (GSDMC) play a role in gastrointestinal health. Nitish et al.^89^ demonstrated that GSDMB is highly expressed and localizes in intestinal epithelial cells (IECs) of Crohn’s Disease (CD) and ulcerative colitis (UC) patients compared to healthy personnel.^89–91^ They showed that GSDMB full length (FL) promotes cell migration and adhesion, including IEC repair and wound closure by regulating focal adhesion kinase (FAK) phosphorylation through platelet-derived growth factor subunit (PDGFA).^89^ Whereas, GSDMB deficiency results in dysregulated epithelial wound repair.^89–91^ In addition, GSDMC, GSDMC2, GSDMC3 and GSDMC4 were upregulated in IECs of mice infected with *N.brasiliensis*, indicating GSDMCs play a role in worm-induced type 2 immunity.^92^

### The role of gasdermin-D in kidney injury

Studies have further shown the effect of GSDMD-mediated pyroptosis in different acute kidney injuries. GSDMD-NT was activated in acute kidney injury (AKI) and renal tubular cell injury induced by the chemotherapeutic agent cisplatin, leading to pyroptosis and renal inflammatory response.

Interestingly, the deletion of GSDMD in AKI mice models alleviated renal inflammation.^93^ Following this study, another group revealed that paricalcitol, a vitamin D receptor agonist, alleviates cisplatin-induced AKI by reducing pyroptosis through downregulation of NFκB κB/ NLRP3/cleaved caspase-1/GSDMD pathway.^60^ Moreoever, GSDMD is associated with AKI induced by radial contrast media such as iopromide.^61^

F. Chen and colleagues^61^ investigated the protective effects of acetylbritannilactone, medicinal herb, in iopromide-induced AKI, which targets NLRP3/ASC/GSDMD and release of IL1β and IL18. To further investigate the activation of GSDMD in kidney injury, N. Maio and colleagues^94^ determined GSDMD activation in tubular epithelial cell pyroptosis, which plays an essential role in acute and chronic kidney injuries. They showed that GSDMD is activated by caspase-11 through cisplatin and ischemia/reperfusion inducing tubular damage and neutrophil infiltration.^94^ In addition, GSDMD-NT activation also results in membrane pore formation secreting urinary IL18.^94^ With that, another group further elucidated the mechanism of tubular epithelial cell pyroptosis in I/R induced acute kidney injury.^30^ They revealed Tisp40 involved in phosphorylation NFκB p65 leads to overexpression of tubular epithelial cell pyroptosis by targeting NLRP3/cleaved caspase-1/GSDMD-NT in I/R-induced kidney injury.^30^ These findings show that inhibition of NFκB using parthenolide, and Tisp40 deficiency reduces renal damage. Therefore, they conclude that Tisp40 regulates GSDMD-mediated tubular endothelial cell pyroptosis through NFκB p65 phosphorylation.^30^

### Gasdermin-D in Autoimmune Disease

Emerging insight highlights the role of GSDMD-mediated pyroptosis in autoimmune diseases. GSDMD activation and inhibition have different effects on autoimmune diseases. For example, GSDMD deficiency in Lupus enhances systemic autoimmunity. Thus, GSDMD is essential in regulating Lupus’s immunological dysfunction.^95^ In contrast, GSDMD deficiency attenuates the pathogenesis of osteoarthritis but does not inhibit the formation of autoantibody-immune complexes.^96^

GSDMD has recently found to play a protective role in autoimmune hepatitis (AIH), with GSDMD depletion exacerbated liver injury, intestinal barrier damage after concanavalin (ConA) injection to induce AIH.^97^ These studies conclude that GSDMD regulation is essential in maintaining immunological homeostasis. Furthermore, aberrant inflammasome activation causes different autoinflammatory responses. More studies are needed to investigate the role of GSDMD in autoimmunity.

## Summary and perspective

**Figure 1.**
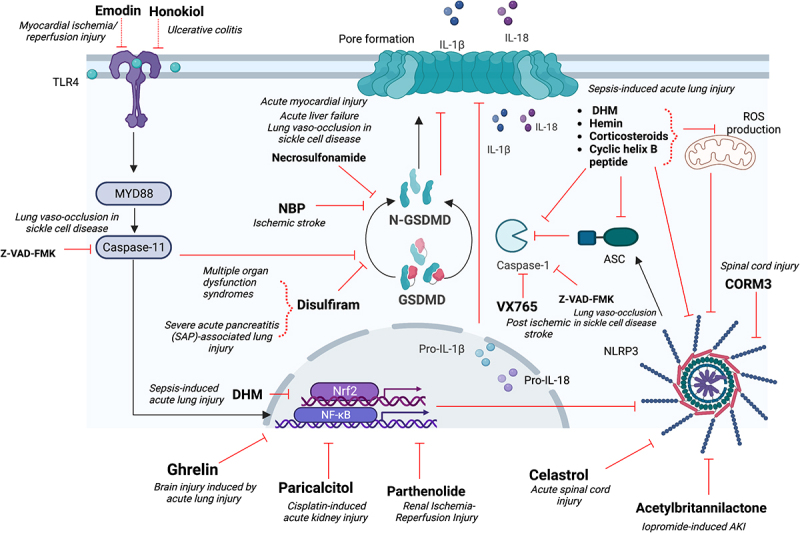
GSDMD contributes to organ injury regulation through different pathways: caspase-1, ASC and NLRP3; TLR4-NFκB pathway; Gasdermin-D cleavage, IL1β and IL18 release.

GSDMD-mediated pyroptosis plays a pivotal role in inflammatory diseases resulting in organ injury, including but not limited to the liver, brain, heart, lungs, gastrointestinal tract, and kidneys. Therefore, many studies reveal GSDMD as a potential therapeutic target. They targeted GSDMD through specific inhibition or inhibition of its upstream pathways, specifically NLRP3 inflammasome or cleaved caspase-1/11/4-5 (Figure 1). GSDMD is part of the Gasdermin (GSDM) family, composed of six members, GSDMA, GSDMB, GSDMC, GSDMD, GSDME/DNFA5, and PVJK/GSDMF.^5^

All other members are equally important and play significant roles in cell death pathways. For example, GSDME is a vital regulator in necrosis, where it plays a critical role in tumor suppression.^98–100^ GSDME and GSDMF were previously identified as DNFA5 and PVJK/DFNB59, respectively, where a mutation in those genes cause age-related hearing loss.^6, 7^ GSDMB is highly expressed in lung epithelium in asthma patients and gut epithelium in inflammatory bowel disease patients;^89, 91, 101^ thus, it plays a pivotal role in lung and intestinal injuries. In addition, GSDMA also contributes to inflammatory bowel disease.^102^ GSDMC is identified as a prognostic factor in lung adenocarcinoma.^89^ In addition, J. Zhang and colleagues identified caspase-8 mediated cleavage of GSDMC in pyroptosis as a potential therapeutic target for tumor progression.^103^

GSDMD has been the most extensively studied protein of the GSDM family due to its involvement in inflammatory regulation and homeostasis. Yet, there are limited number of GSDMD inhibitors going into clinical trials. Disulfiram is the only FDA-approved drug and potent GSDMD-NT inhibitor; however, it is only approved for alcoholism.^104^ Future studies need to investigate the role of disulfiram and adapt it to inflammatory diseases and organ injury.
